# IoT-Stream: A Lightweight Ontology for Internet of Things Data Streams and Its Use with Data Analytics and Event Detection Services †

**DOI:** 10.3390/s20040953

**Published:** 2020-02-11

**Authors:** Tarek Elsaleh, Shirin Enshaeifar, Roonak Rezvani, Sahr Thomas Acton, Valentinas Janeiko, Maria Bermudez-Edo

**Affiliations:** 1 Centre for Vision, Speech and Signal Processing, University of Surrey, Guildford GU2 7XH, UK; s.enshaeifar@surrey.ac.uk (S.E.); r.rezvani@surrey.ac.uk (R.R.); v.janeiko@surrey.ac.uk (V.J.); 2 School of Computer Science, University of St Andrews, St Andrews KY16 9SX, UK; sta1@st-andrews.ac.uk; 3 Software Engineering Department, University of Granada, 18012 Granada, Spain; mbe@ugr.es

**Keywords:** IoT, data model, ontology, data stream, semantic model, linked data

## Abstract

With the proliferation of sensors and IoT technologies, stream data are increasingly stored and analysed, but rarely combined, due to the heterogeneity of sources and technologies. Semantics are increasingly used to share sensory data, but not so much for annotating stream data. Semantic models for stream annotation are scarce, as generally, semantics are heavy to process and not ideal for Internet of Things (IoT) environments, where the data are frequently updated. We present a light model to semantically annotate streams, IoT-Stream. It takes advantage of common knowledge sharing of the semantics, but keeping the inferences and queries simple. Furthermore, we present a system architecture to demonstrate the adoption the semantic model, and provide examples of instantiation of the system for different use cases. The system architecture is based on commonly used architectures in the field of IoT, such as web services, microservices and middleware. Our system approach includes the semantic annotations that take place in the pipeline of IoT services and sensory data analytics. It includes modules needed to annotate, consume, and query data annotated with IoT-Stream. In addition to this, we present tools that could be used in conjunction to the IoT-Stream model and facilitate the use of semantics in IoT.

## 1. Introduction

Internet of Things (IoT) has introduced radical changes in the way data are processed. The amount of IoT data, the velocity of change, variety of sources and formats of said IoT data implies new challenges to process and interoperate between heterogeneous data sources and formats. Especially in large-scale IoT solutions, new solutions are being proposed in order to deal with the big IoT data.

Semantics is one the most used solutions to overcome the heterogeneity of IoT data sources and formats. Semantics provide common information models with which the services using heterogeneous sources of information could interoperate using the same concepts and relationships between concepts. Examples of these solutions include information models describing IoT devices, services, types, and units of data, etc., such as the models in [1,2,3,4].

Some years ago, the main bout of the ontologies were to describe in detail the real world, annotating as much information as possible to represent the real world in the ontology. However, with the growth of the number of sensors and data, the time to annotate and query the ontologies has become a bottleneck in the (quasi) real-time processing of data coming from the IoT environments. Therefore, having lightweight models with a minimum number of concepts and relationships between concepts, enough to allow the regular searches and crawling of IoT data streams, will improve the processing time of the IoT data. In the last few years, some researchers have conceived and applied the idea of lightweight information models in the field of IoT, such as IoT-Lite [5], and the Semantic Sensor Network model (SSN) [6], which is a standard de-facto for modelling sensors. SSN has recently published a lightweight ontology, SOSA (Sensor, Observation, Sample and Actuation) [7], which has become the core ontology of the new version of the compendium of ontologies, SSN. However, the lightweight models mentioned, IoT-Lite and SOSA, are centred around devices, but do not pay enough attention to the IoT data streams. For stream annotation, there is a lack of lightweight models. Some models provide detailed stream annotations, such as SAO (Stream Annotation Ontology) [8]. However, these detailed annotations added some delay in processing time during data stream acquisition and processing, making it difficult to operate with (near) real-time applications.

In light of this, we propose IoT-Stream, a lightweight semantic model for stream data annotation, which is centred around the concept of an IoT-Stream, and extends the SOSA ontology (and by extension SSN). The main idea behind IoT-Stream is the simplicity of the information model, and especially the individual streams, which are the heavier part of the annotations, as they represent most of the information annotated. Therefore, each stream observation in our proposal is composed of only a value and a timestamp. Hence, we have segregated all the metadata needed for searching and crawling purposes, but not required for (quasi) real-time data processing. Furthermore, our proposal allows for annotating raw data as well as processed data, and both of them could be annotated as streams and could be kept lightweight. For instance, a sensor generates one data value per minute, and we could annotate individually this raw data. However, the applications using the information model could not require such fine granularity and using data every ten minutes will be enough. In that case, we could apply some data mining algorithms to aggregate the raw data in windows of ten minutes. For example, we could use SAX (Symbolic Aggregate Approximation) to aggregate the raw data, and annotate with IoT-Stream the processed data stream. Both the raw stream and the processed stream are lightweight because both streams annotate only the observation value and either the timestamp (for the raw data stream) or the window interval (for the processed data stream). For searching/crawling purposes, we also attached metadata outside of the stream observations such as applied algorithm and its parameters with their values.

Having good semantic models and creating ontologies is not enough. Semantics are not the end-product, they are only models to be used by applications, so the focus of the ontology design should be on extending the models with effective methods, tools, and APIs (Application Programming Interfaces) to handle and process the ontologies. Queries and analytics should be able to effectively use these semantics [9]. In order to facilitate the adoption of semantics, we also proposed a reference system, with the necessary entities to annotate, analyze, and query the stream data. Later in the paper, we also describe some successful use-cases, tools, and applications using IoT-Stream and the system instantiation used in each of the use cases.

The remainder of the paper is organized as follows. Section 2 describes the related work. Section 3 introduces the IoT-Stream ontology, and how it was created and why. Section 4 provides an example of a system architecture for dealing with annotated data with our model in IoT scenarios. This section will help developers to adopt and reproduce our ontology and scenarios or similar ones. Section 5 introduces several case scenarios that have been successfully used and that illustrates the semantic annotation of sensor stream data with our model. Section 6 details several applications which could be used in conjunction with our model and that could help in the adoption and automation of the stream processing. Finally, Section 7 concludes the paper and describes the future work.

## 2. Related Work

Semantic models representing stream annotations are scarce. To represent IoT data streams, we need concepts that represent devices, location, time, quantity units, values, and streams. In the description of devices, there are some models to represent sensors and their observations. The most representative model is the SSN ontology which describes sensors with their properties, systems, deployments, stimuli, and observations [6]. The SOSA ontology is a lightweight core for SSN that provides concepts for sensors, observation values, and features of interest [7]. IoT-Lite is another lightweight model for IoT concepts with the aim of fast annotation, processing, and semantic querying time. IoT-Lite was inspired by the IoT-A reference model [10] which defined core concepts for the IoT, namely Resources, Entities, and Services. It instantiates and extends Device and Sensor concepts from SSN [11]. The focus of IoT-Lite, SOSA, and SSN is more on sensing devices and is appropriate for sensor discovery, lacking specific concepts for stream annotation and aggregation. For example, Le-Phuoc et al. [12] create a graph of things with spatial and temporal annotations using SSN and in a use case representing flights in a map to annotate streams. However, they have just annotated individual values, not aggregated values. Furthermore, each annotation has several classes and properties; hence, the annotation and querying require a processing time which could be improved with a lightweight annotation focusing on stream requirements.

There are some location models, such as Geo (https://www.w3.org/2003/01/geo/) that helps in searching for IoT devices. Geo is a popular model that represents location data in RDF, and it does not try to tackle many of the matters covered in the professional Geographic Information System (GIS) world. Instead, the ontology offers just a few basic simple terms that can be used in RDF when there is a need to describe latitudes, longitudes, and altitudes. The use of RDF as a carrier for latitude, longitude, and altitude simplifies the capability for cross-domain data mixing, as well as describing entities that are positioned on the map (e.g., carrying out geospatial queries for Sensors, Deployments, Platforms, or Systems). GeoSPARQL is a standard for the representation and querying of geospatial linked data for the Semantic Web from the Open Geospatial Consortium (OGC) [13]. GeoSPARQL defines location-related concepts to facilitate sensor discovery as per spatial requirements. GeoJSON (https://tools.ietf.org/html/rfc7946) is a geospatial data interchange format based on JSON. It describes numerous types of JSON objects and the way they are joined to represent data about geographic features, their properties, and their spatial extents. GeoJSON supports a range of geometry types ranging from Point, LineString, Polygon, MultiPoint, MultiLineString, and MultiPolygon.

The Time ontology (https://www.w3.org/TR/2017/REC-owl-time-20171019/) is a well-known and widely used semantic model to represent time. It has a vocabulary for representing information about topological (ordering) relations, duration, and temporal position (i.e., date–time information). Time can be expressed using conventional clock, Unix-time, geologic time, and other reference systems. For duration, it can also use different systems—for example, Gregorian calendar as in [14]; this ontology has been extended in the last versions with various temporal concepts, such as instants and intervals, and interval relationships. These concepts were present as well in the TimeLine ontology [15], together with concepts of timelines (e.g., universal or discrete). These time ontologies have been used to annotate streams and have inspired the querying of stream data.

There are also some ontologies to provide quantities, units, dimensions and values. The QU ontology (https://www.w3.org/2005/Incubator/ssn/ssnx/qu/qu-rec20.html) is one of the well-known ontologies in this field. Qu ontology has been developed to support different Systems Modelling Language (SysML) users [16].

Another important aspect of stream data annotation is the Quality of Information (QoI) because faulty data can have costly consequences [17]. When talking about Quality of Information, categories or metrics are important to describe the details. There are five common metrics: Completeness, Correctness, Concordance, Currency, Plausibility [18], and Security [19]. In the CityPulse project (http://www.ict-citypulse.eu/page/), they extend the ontology and used five categories; Timeliness, Cost, Accuracy, Communication, and Security, each with a collection of sub-metrics. The major problem in the model is lack of ground truth in Correctness which has been addressed in [20] with spatio-temporal, causality, and outcome evaluation.

The previously mentioned ontologies could help in the annotation of stream data, but do not tackle all the concepts needed for this type of data, and missed essential concepts, such as aggregation of streams that could leverage the processing time when querying stream data.

There are few ontologies representing stream data. One representative is SAO. SAO has been built on top of some well-known ontologies to represent IoT data streams: TimeLine [15], PROV-O [21], SSN [6], and Event Ontology [15]. StreamData, StreamEvent, StreamAnalysis, Observation, Sensor, and Segment concepts enable this ontology to describe temporal concepts accurately. With StreamData class, SAO can provide a stream data as a temporal point or segment and it describes the output of the observation as an event with StreamEvent class [8]. We propose to enhance SAO by reducing the triples needed for querying the stream data.

Recently, the RDF Stream Processing Community Group has attempted to define a common model for producing, transmitting, and continuously querying RDF Streams. Their focus is on extending RDF and SPARQL to represent and query stream data. Their solution is based heavily in previous solutions for querying stream data, such as SPARQLstream [22,23], C-SPARQL [24], EP-SPARQL [25], Instants [26], CQUEL [27], or STARQL [28] that allows for a uniform querying of both streaming and static data. Although these extensions of SPARQL for streams are highly appealing and need to be used by any stream annotation model, like the one we propose here, they do not focus on the annotation of the streams, but only on the querying. In the field of stream annotation models, the RDF Stream Processing Community Group is studying how to modify RDF to facilitate the representation of streams. Thus far, what they have published is to represent an RDF stream as a sequence of time-annotated graphs <g [t]>, where g is an RDF graph and t is a timestamp [29]. This approach has already been used by Siemens and in the European project Optique to annotate streams [30,31], using the query language STARQL. The Optique platform is a complete platform, including a deployment module, BootOX for ontology and mapping bootstrapping, the query language STARQL, a backend, ExaStream, which process the data, and a query interface, OptiqueVQS [32], allowing to write queries for non-experts, without knowledge of the query syntax. This work is an excellent solution, which can be used together with our light ontology solution to improve the performance of the annotations and queries. However, this solution involves the modification of the standard RDF, and therefore the adaptation of the involved reasoners (including triple stores) and tools, which means that at the present moment some reasoners would not work with this RDF notation. To take advantage of the current tools, we opt for a solution that does not imply the modification of RDF—although in the future our proposal could be easily modified to accept the new version of RDF notation upon its acceptance as standard, and our model could support both standards.

## 3. IoT-Stream Ontology

### 3.1. Vision, Design, and Best Practices

**Figure 1:**
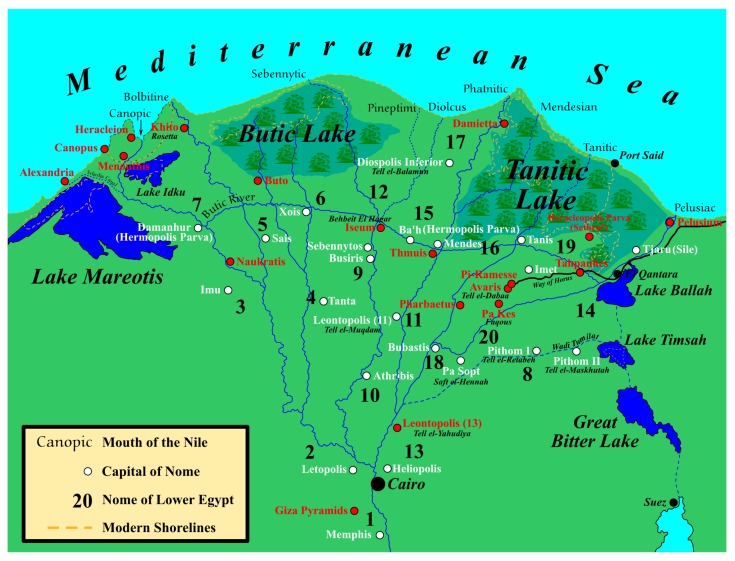
An Analogy of Internet of Things (IoT) stream data as waterways derived from other waterways.

The vision for an ontology for IoT stream data can be described through an analogy where stream data can be compared to rivers and canals as shown in Figure 1 (https://commons.wikimedia.org/wiki/File:Map_of_Lower_Egypt.svg). As can be seen from the bottom of the figure, these waterways can branch out and feed to one or more other streams, all of which are destined to a (data) lake or sea. Streams that are created can be a result of some form of processing, such as in the case of water supply and sewage systems. During processing, analysis can be applied to detect changes, abnormalities, patterns of interest, events, or even some form of treatment, as is the case with data streams.

To reflect this into the design of the ontology, concepts of stream derivation and analysis need to be defined. Since IoT data streams are expected to produce observations on the scale of big data, it is imperative to maintain a light approach to defining the stream observations concepts, and to segregate them from descriptions relating to the IoT stream as a whole. For an ontology to be effective for adoption, the development of an ontology must be have a foundation with well-established best practices, such as those defined in the ontology creation guide [33]. Here, the first consideration is to define the ontology’s domain and scope, which in this case revolves around the concept of data streams produced by IoT sources, with a focus on concepts to support data analysis, event detection and provenance. From extensive work on previous projects focusing on defining concepts for IoT entities and data, such as in [8,34,35,36], a common challenge was a trade-off between expressiveness in data annotation, persistence size, and efficiency in querying. For IoT stream data and analysis, the scope must be focused on how their consumption will work in a scalable system. The next consideration is to adopt existing concepts from other ontologies that can enrich the model with metadata that is useful for describing the main concept—in this case, the IoT Stream. Here, concepts relating to space, time, theme, device association, service exposure, and data quality are highly relevant, which are available and well-established in the IoT community. Its adoption is explained in Section 3.3. The third consideration is what terms are to captured in the ontology. Regarding streams and stream data, the time instant or interval at which the observation was made is important. Regarding the value, it should be simple but flexible as observation formats can vary between systems. For terms relating to data analytics applied to streams, terms such the methods and parameters used in a particular technique are needed. An important output of analysis are events and alerts that are detected from the streams, and hence labels and the temporal aspects should be captured. The fourth consideration is to adopt a hierarchy for the classes defined. As the intention for this ontology is to be lightweight, the only focus here would be on the stream observation. The SSN ontology provides a concept for observations and meets the basic requirements defined earlier, and so it has been adopted and extended to cater to the nature of stream observations, as explained in Section 3.2.

Another aspect to take into account is that the ontology should reflect principles adopted in ontologies that have been highlighted by standardization bodies for the application of best practices, such as the W3C Good Ontologies (http://www.w3.org/wiki/Good_Ontologies). These principles relate to the quality of the associated documentation of the ontology, and that it is an Internationalized Resource Identifier (IRI) is dereferenced. It should also demonstrate adoption by data producers for annotation, and is supported by existing tools, which is presented in Section 5 and Section 6. Once all of these aspects are applied, an information model for IoT stream data can be formulated.

### 3.2. Information Model

**Figure 2:**
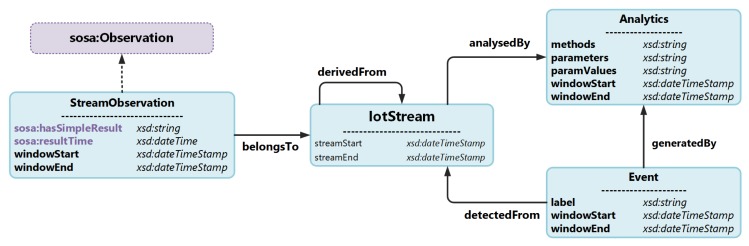
IoT-Stream classes and properties.

The principal information model focuses on modelling stream observations, its analysis and events that are detected from it, which are captured in four classes. These classes reflect the concepts of an IotStream, a StreamObservation, an Analytics process and an Event. As depicted in Figure 2, the central class that the other classes directly link to is the IotStream. This abstraction represents a data stream originating from an IoT data source. It has annotation properties that capture the lifetime of the IoT data stream that would mainly be used for reference rather than actual consumption by an application. This annotation properties are streamStart and streamEnds. As mentioned earlier, the vision of an IotStream is that, like waterways, they can branched off other streams, and hence be derivedFrom other IotStreams. This can be a result of some form of processing of the IoTStream that is being dervivedFrom, such as filtering, re-sampling, or aggregation.

The class that would be of main interest and consumption is the StreamObservation that belongsTo it. The value of an stream observation can take several forms. The first being atomic, as a data point. Second, it could have a bulk representation as a vector of data points. The stream observation also contains an instantaneous timestamp which keeps a record of when the observation was captured. When considering the reuse of other popular ontologies, the SOSA ontology provides a class that meets the requirements for capturing a sensor observation, which is the sosa:Observation class and it datatype properties, sosa:hasSimpleResult and sosa:resultTime. Another requirement is to be able to capture the temporal aspect for an observation as an interval or window. The sosa:Observation provides the datatype property sosa:phenomenonTime for this purpose, and links to the time:TemporalEntity class from the popular Time Ontology—although the issue here is the complexity involved in representing the window and linking it to the observation. As one of the main aims of this ontology is to inhibit a lightweight manifestation, the sosa:Observation class has been extended with a subclass, StreamObservation, to include direct datatype properties for representing temporal windows. These are captured in the data properties windowStart and windowEnd, which represent the start and the end of the window, respectively. Another important consideration for StreamObservations is to segregate it from the rest of the metadata, since the number of instances created would be significantly higher in proportion with respect to the number of IotStreams, and, for this reason, the extension from the instantiation of the sosa:Observation class is intentionally kept to a minimum.

StreamObservations that belongToIotStreams can be either the output of sensor readings, or the output of an Analytics process. In the case where IotStreams are analysedBy a data analytics process, the Analytics class captures the methods from data analysis techniques applied on the IotStream. It can be a single process or a cascade of processes, and hence is represented as a vector string with the data properties methods parameters and paramValues. The data property methods captures the different methods or algorithms with which the stream has been analysed. The data property parameters set for these methods are also captured as a vector string, whereby the first element in the methods vector corresponds with the first element in the parameter vector. It is worth noting that methods can of course set multiple parameters, and so the corresponding element in the parameters data property can be an array of parameters in itself as well. For each parameter, the values that are set are also captured in the data property paramValues. An Analytics process that is applied to an IotStream can possibly be active during a temporal window with the lifetime of an IotStream. Hence, the data properties windowStart and windowEnd are used for this case. The Analytics class can also be exclusively used to define the data analysis process that is used to generate Events that are detectedFrom an IotStream, which would be applicable in cases such as classification or clustering. The Event contains properties that capture the data property label that is used to describe the Event, and the temporal interval the Event is also relevant. This can be useful information for data scientists to understand how the Event was generated.

### 3.3. Linked Models

As mentioned earlier regarding best practices with respect to ontology reuse, the information model adopts several concepts which are regarded as core attributes to provide real-world context to the IoT stream.

The first relates to the spatial attributes of the IotStream. The W3C Geo ontology provides a set of basic concepts that represents the location of an entity. The main concept of interest is the geo:Point which contains geospatial properties (Latitude, Longitude, and Altitude). The IoT-lite ontology [11] extends the properties to include the relative location and relative altitude (e.g., level 1). To maintain the historical context for StreamObservations, especially in the case of mobility, a geo:Point can be linked to each StreamObservation. The IotStream can also be associated with a defined coverage area where it is also relevant. The iot-lite:Coverage concept can be used for simple coverage definitions, and GeoSPARQL [13], which is a well-established ontology for spatial attributes, can be used for more complex area definitions.

The next subset of adopted concepts relate to the generating source of the IotStream and the phenomena and measurement of its observations. As streams in the real-world are generated by sensors, the SOSA [7] Sensor concept is linked. Through the object properties defined by IoT-Lite, the QU ontology’s qu:QuantityKind and qu:Unit concepts are also linked. For actual instantiations for these concepts, taxonomies such as QU-rec [16] and M3-lite [36] can be used.

Even though it is the sensor device the generates the IoT stream, through the Internet, the stream data are usually provided by a TCP/IP Application Layer Service. IoT-Lite provides a iot-lite:Service class that contains fields relating the service’s endpoint address, the type of interface, and the link to the interface’s description, which provides details on how to interact with the service.

**Figure 3:**
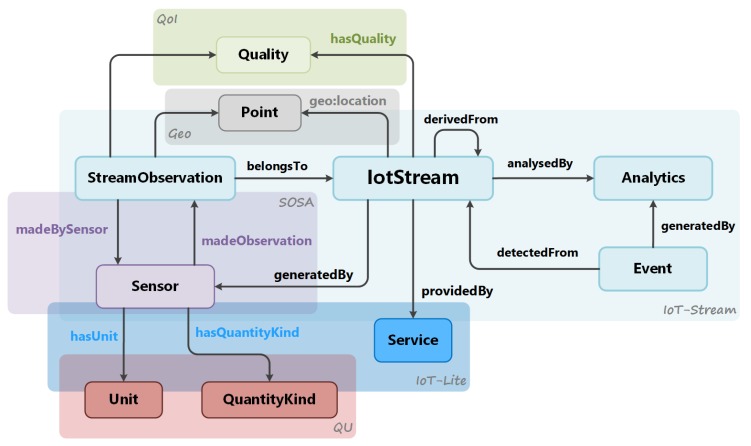
IoT-Stream linked with the main ontologies.

Finally, throughout the lifetime of an IotStream, the quality of the stream observations can change over time. For data analysis, knowledge of quality is very important so that adaptive measures can be applied when needed. The Quality of Information (QoI) ontology provides the concept qoi:Quality that has subclasses which focus on a particular aspect of quality, such as qoi:Timeliness and qoi:Completeness of observations. Figure 3 illustrates how IoT-Stream is linked to these external concepts, and Table 1 lists the namespaces of the linked ontologies and their preferred prefixes.

### 3.4. Model Navigation and Querying

**Figure 4:**
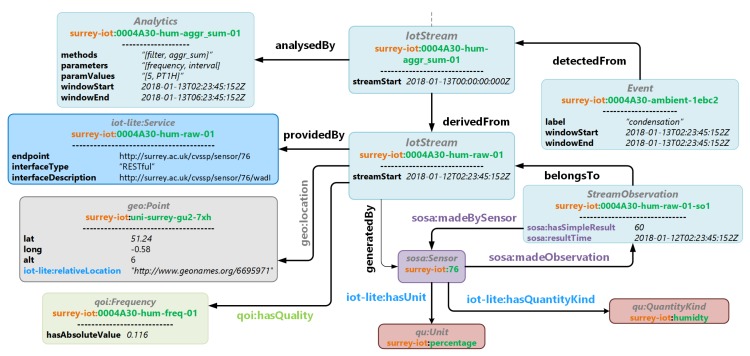
An instantiation of IoT-Stream.

It is important to consider how a graph based on IoT-Stream is to be navigated or queried. The IotStream concept serves as a root node in a graph which directly links to the most significant metadata in the context of queries relating to IoT data. This approach enables simpler queries as graph traversing is kept to a minimum. Figure 4 illustrates an instance of an IotStream. Here, the IotStream is generatedBy a sosa:Sensor that sosa:madeObservations that measure the qu:QuantityKind for humidity with a qu:Unit of percentage. StreamObservations that belongTo an IotStream are atomically annotated. The IotStream’s geo:location is a geo:Point with absolute and iot-lite:relativeLocations for the University of Surrey. The IotStream is providedBy a iot-lite:Service with an iot-lite:endpoint with a RESTful iot-lite:interfaceType. Information about the qoi:Frequency of the IotStream is also linked through a monitoring process. A second IotStream is derivedFrom it through an Analytics process that involves filtering and aggregation. An Event indicating "condensation" is detectedFrom the second IotStream.

### 3.5. Ontology Metrics and Documentation

The principal information model for IoT-Stream has four classes, five object properties, and eight data properties, as shown in Figure 2. It also borrows some classes and other properties from other information models. The global model with core and borrowed items have 21 classes, 14 object properties, 13 data properties, 274 axioms, 60 logical axioms and 73 declaration axioms, nine subclasses, two disjoint classes, one subobject property, one inverse object property, 13 domain object properties, 14 range object properties, one subdata property, 10 domain data properties, nine range data properties, 127 annotation assertions, 10 domain annotation properties, and four range annotation properties.

The ontology is publicly available (http://iot.ee.surrey.ac.uk/iot-crawler/ontology/iot-stream) in several RDF variants with dereferenceable concepts. The public URL also includes documentation with graphs and a description of the ontology, to facilitate the adoption. It can also be reached using its derefenceable URI: http:/purl.org/iot/ontology/iot-stream#.

## 4. System Architecture and Data Management

When defining a new ontology for IoT, it is essential to demonstrate how it can be used for IoT-oriented systems. This section defines what system entities are needed, and approaches can be taken to exploit IoT-Stream with respect to requirements relating to annotation, publishing, persisting, querying, and subscribing to IoTStreams.

### 4.1. System Entities

**Figure 5:**
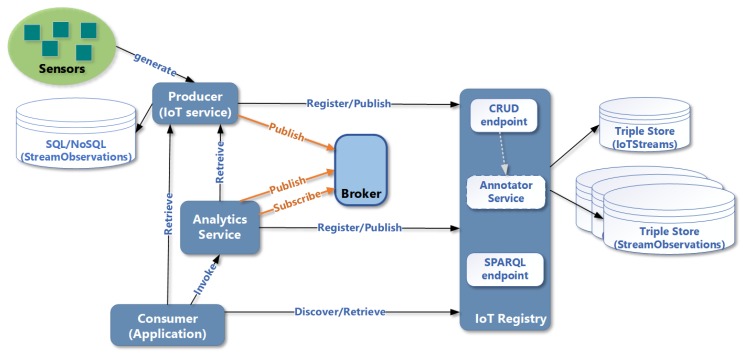
System entities and interactions for IoT-Stream adoption.

For a system to support the adoption of IoT-Stream, the essential system entities required, as depicted in Figure 5, would be:

- **Registry**: primarily responsible for storing information about an IotStream in a triple store and exposing a SPARQL endpoint for handling queries. It could also be employed by an IotStream producer to store StreamObservations, hence undertaking the role of the IoT service.
- **Producer**: responsible for registering IotStreams and publishing the StreamObservations generated from its sensors. If it is capable of storing and exposing StreamObservations, it can act as the IoT service.
- **Consumer**: an application or service that discovers IoTStreams via the registry and consumes StreamObservations using the designated IoT service. In the context of data analytics, the consumer can consume analysed data for business intelligence or pre-processing of StreamObservations (such as aggregation or filtering).
- **Broker**: an alternative to persisting StreamObservations, in which Consumers and Services can subscribe to real-time StreamObservations published by a Producer to the stream data broker. In this case, persistence is up to the Consumer.
- **Analytics Service**: employed by a Consumer or Producer to consume or generate analysed IoTStreams, by applying specific data analysis techniques with either a specific method or a set of methods. This could be part of the Consumer’s internal system or an external microservice that focuses on a particular type of analytics.

### 4.2. Data Flow within System Entities

**Figure 6:**
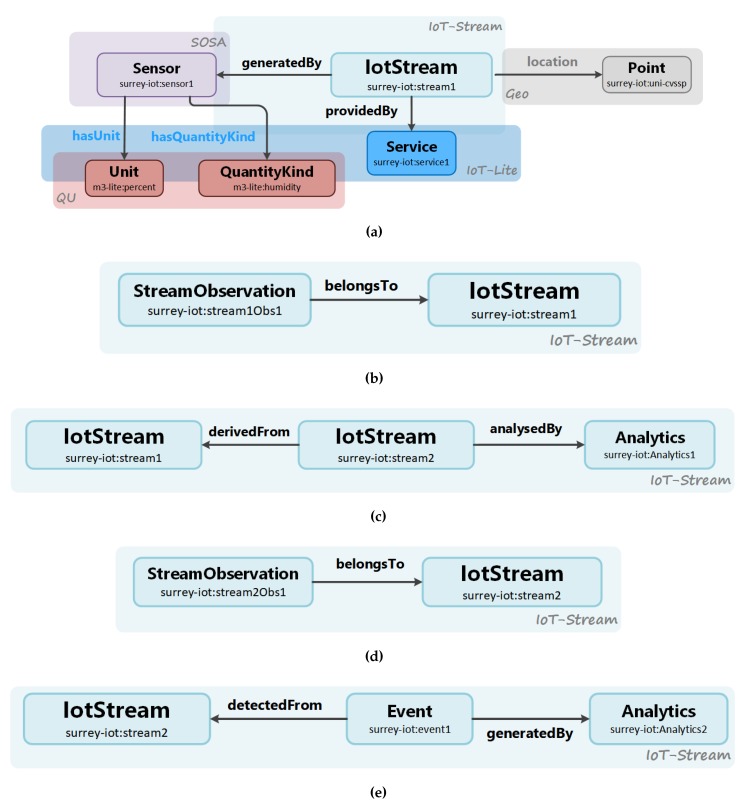
Ontology concepts used in data flow between system entities: (**a**) A Producer registering an IotStream. (**b**) A Producer publishing a StreamObservation. (**c**) A Consumer registering an IotStream derived from an IotStream analysed by an Analytics Service. (**d**) An Analytics Service publishing an analysed StreamObservation. (**e**) Another Analytics Service publishing a generated Event.

A *Producer* would normally have a proprietary information model for modelling its sensor metadata and data, which is appropriate for use within its local system. To share data externally, the *Producer* would need to register and publish its data into an externally reachable *Registry*. The *Registry* can act as either two roles; the first being a directory and the second, as a repository. As a directory, the *Registry* will only store information about the streams and not the stream observations in an RDF store. Here, the *Producer* transforms it proprietary descriptions and instantiates an IotStream using a semantic annotator. An IotStream individual is created, with the streamStart time. It also creates a sosa:Sensor individual with the corresponding qu:QuantityKind and qu:Unit. It also appends the location of the IotStream. In addition, lastly, the iot:Service which defines the endpoint and interface the StreamObservations will be providedby (Figure 6a). The service would either be hosted in the *Producer’s* domain or at a delegated broker. If the *Registry* assumes the role of a repository, then *Producers* can delegate the Service to the *Registry* itself. The *Producer* then starts publishing StreamObservations to the delegated IoT service (Figure 6b), which then *Consumers* will be able to retrieve or subscribe to. If the *Consumer* will not consume the StreamObservations as they are originally generated by the Sensors, it can employ an *Analytics Service* to retrieve or subscribe to them. Here, the *Consumer* registers a new IotStream with the *Registry* (Figure 6c), and submits to the *Analytics Service* the IoT Service to obtain StreamObservations from, and the callback Service to publish the new analysed StreamObservations (Figure 6d). The *Consumer* can employ another *Analytics Service* to consume the analysed IotStream to detect Events (Figure 6e).

### 4.3. Annotation Considerations

The ontology has been designed to allow flexibility in the way stream observations are annotated and stored. Annotation can be done on the atomic level, whereby an observation corresponds to a data point or it could be non-atomic (bulk), whereby observations correspond to a window of data points represented as a vector string. Alternatively, the service that provides the streams is only annotated, and streams are kept without semantic annotation. In this case, the StreamObservations are retrieved from an external IoT service that provides the StreamObservations belonging to an IoTStream, as demonstrated by [37]. This IoT service may serve StreamObservations using simple data formats, e.g., CSV or JSON. Otherwise, if the Consumer requires them in RDF, then it can utilize an “on-the-fly” semantic annotator for providing StreamObservations in an RDF variant upon request, such as Turtle or JSON-LD.

With these three annotation approaches, each comes with their trade-offs. The atomic approach to annotating would provide less processing burden at the *Consumer* side when querying but more on the *Registry* to process the query. The bulk approach would introduce more processing at the *Consumer* side for parsing StreamObservations, and would relieve the Registry from searching for more instances. The final approach would require the *Consumer* to fetch StreamObservations from another endpoint, which require the Consumer to make a second request with a different interface, but will relieve the *Registry* from storing StreamObservations altogether.

In the case of a *Producer* being hosted on a constrained device, a dedicated annotator service outside the device can be employed. This service would typically require the stream observations in a pre-defined format. For example, a dedicated annotator could transform CSV format into JSON-LD that follows the IoT-Stream model.

When IotStreams are derived from another IotStream, the Analytics applied, i.e., the methods and their corresponding parameters need to be declared and annotated. A practical approach is to apply the convention defined in [38], whereby methods and parameters are captured each in an array, with the first index in the methods array corresponding to the first index of the parameters array, and so on. Another issue that arises is the naming convention to adopt for the methods and parameters. The Python scikit-learn [39] module has become the most popular package used by data scientists and engineer for analysis, so its vocabulary could be used as a reference for annotating Analytics processes.

### 4.4. Storage and Querying

Triple stores are suitable for storing finite information about entities, but not so for time-series data that are associated with them. In the case of time-series data, as more data are accumulated to a dataset, triple stores begin to struggle to respond to queries within reasonable periods, for example as evaluated in [40]. If an atomic annotation is required, then a StreamObservation dataset needs to be separated from the rest of the metadata, except its link to the IoTStream it belongsTo. In this case, a SPARQL federated query can be used to discover IoTStreams and in turn retrieve observations from another dataset using the iot-lite:endpoint. If StreamObservation retrieval is done without a SPARQL endpoint, the iot-lite:interfaceDescription can also be retrieved to know what parameters to pass for retrieving an instantaneous observation or a set of observations within a defined window. For example, in a RESTful services, a WADL would typically be used for this purpose.

Another aspect to consider is the variability of metadata associated with an IotStream, such as location and quality. In the case of location, if the sensor generating the IotStream is attached to a mobile entity, then location information would need to be captured and linked to each StreamObservation. A query in this case would need to include a check on the mobility status of the sensor using iot-lite:isMobile. The IotStream itself would only have the current location information linked to it. For quality of information, metrics such as qoi:Timeliness can change during the lifetime of an IotStream, whereby a sensor experiences computational or connectivity issues whether internally, i.e., on-device, or externally caused by an intermediary node such as a gateway). In these cases, new QoI instances would need to be stored with each StreamObservation, or linked to each.

## 5. Use Cases

An illustrative approach to demonstrate how the IoT-Stream ontology and system architectures that adopt it is to apply it in different use cases. Here, smart cities and smart living domains are taken as examples.

### 5.1. Smart City Traffic and Environment

**Figure 7:**
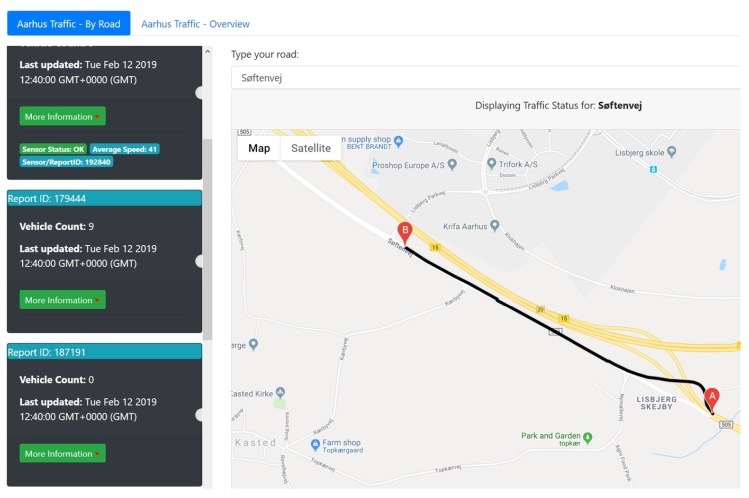
Smart city traffic analysis.

As part of enabling a city to become smart and drive its digital economy through research and industrial innovation, the provision of open data regarding different aspects of these growing cities that have an impact on the citizen is vital to enable a sustainable, healthy, secure, and prosperous environment. Cities around the world are increasingly adopting this vision. Some provide historical data which is useful for discovering trends and future predictions, and some go the step further to provide real-time sensory data, such as the case with cities such as London (https://www.londonair.org.uk), Brussels (http://data-mobility.brussels/), and Aarhus (https://portal.opendata.dk/). In the municipality of Aarhus, data feeds relating to traffic are captured live and published on their public CKAN repository every five minutes. The capturing of observations is enabled through devices located around the city that sense Bluetooth signals from passing vehicles. For each vehicle, the system records the starting and ending detection points. The resultant route is then used to calculate the speed and then averaged over multiple vehicles. In addition, it records a count of vehicles for every five minutes. Metadata relating to sensor identification, calibration, location, and place is also provided. Here, IoT-Stream can be adopted to annotate this data stream as linked data, which can then be used or compared with other traffic-related data streams. In addition to annotating the stream instance, its observations, it can also annotate the Analytics process involved in pre-processing the data stream. Events generated from it such as irregular road conditions relating to periods of unexpected high (or low) traffic can also be captured. The linked ontologies can also be used to capture location information important for geospatial analysis using geo:Point and iot-lite:relativeLocation. Quality metrics such as qoi:Completeness can also be annotated for data monitoring purposes. Figure 7 illustrates visual analysis of the data stream captured using a web application.

The Aarhus open data repository also provides other real-time datasets such as air pollution and wind. These stream observations can also be taken into consideration for analyzing the impact of traffic on the local environment. From the traffic dataset, traffic count and average speed can be selected for traffic analysis. As for the air quality dataset, Particulate Matter (PM) and Nitrogen Dioxide (NO${}_{2}$) could be selected for pattern analysis in air quality. The analytics process could be done in the following manner. In each dataset, the frequency of data generated by sensors is five minutes, and a target is set to represent patterns for every hour. Regarding the hourly pattern representation, the step size in this case would be 12. For analysis, data are split into windows of 12 data points and Lagrangian Pattern Representation (LPR) [41] is performed on each window. The result is hourly patterns of data. After pattern representation, Gaussian Mixture Models (GMM) is used to apply clustering on the patterns, and grouping the patterns in three different clusters. By looking at the cluster’s centres, a label is given to each cluster.

**Figure 8:**
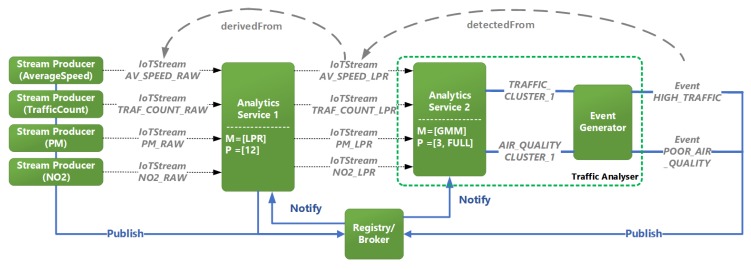
Annotation process for detecting traffic and air quality levels.

Figure 8 shows an excerpt of the instantiation of the system presented in Section 4, and the annotation process which reflects this use case. The blue arrows indicate the actual flow of the stream data, whereas the greyed-out arrows illustrate the eventual flow of the stream. In the Analytics Services, “M” and “P” correspond to the methods and parameters applied on incoming StreamObservations. Here, four IotStream Producers publish StreamObservations to a broker, and two Analytics Services are subscribed to notifications of StreamObservations by the broker. In the case of Analytics Service 1, StreamObservations are received within an hour for traffic and air quality streams are processed using LPR, and the output is annotated with which IotStream instance it was derivedFrom, and is published to the broker. Analytics Service 2 is then notified and applies GMM on the analysed StreamObservations, and are then grouped in the predefined clusters. The output of the clusters are fed to an Event Generator, which generates an annotated Event with label for “HIGH/LOW TRAFFIC” for congestion, or “POOR/GOOD QUALITY” for air quality, and the IotStream instance it was detectedFrom. This output is then published to the broker for any Consumers e.g., Smart City applications are subscribed to it.

### 5.2. Smart Healthy Living for Senior Citizens

**Figure 9:**
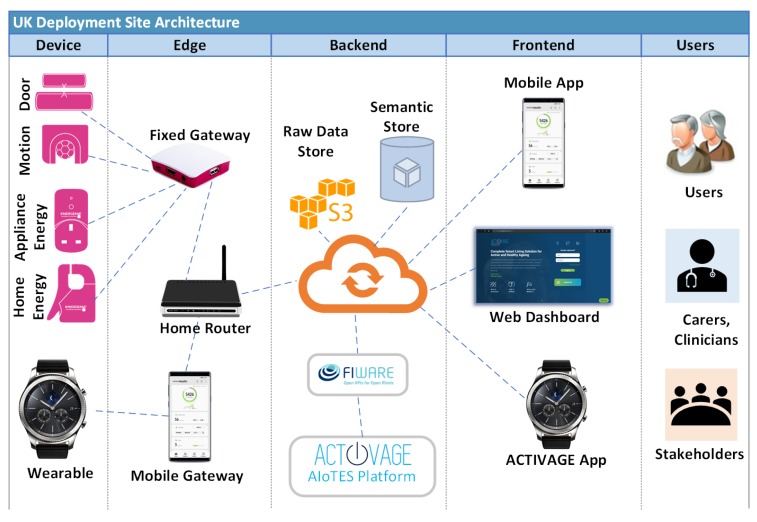
Capturing IoT stream data from Smart Home environments.

The ageing population globally has been growing since 1950 due to increasing life expectancy, and is growing at a faster rate (https://www.un.org/en/sections/issues-depth/ageing/). This has put pressure on governments to increase spending on elderly care. Initiatives have been made to introduce pilot programmes that deploy remote health monitoring technology in homes as a means to support and extend independent living. This is to improve the quality of life for the senior citizen, reduce load on social and health services, and provide clinical and social intervention when needed, and as early as possible. Here, IoT devices play a crucial role in capturing and providing information about the citizen’s wellbeing. A large scale-pilot programme, ACTIVAGE (http://www.activageproject.eu/deployment-sites/Leeds/), has created an IoT-based ecosystem, whereby IoT devices have been deployed in homes for over 7000 users, in multiple deployment sites across Europe. One of the use cases covered by some of the sites is monitoring activities of daily living (ADL). Here, a suite of sensors is deployed in the home, in the form of wearables and mounted devices, which capture physiological observations relating to personal health and ambient environmental conditions. Figure 9 illustrates the system involved. To enable monitoring, data streams of these observations need to be relayed in real time to data analysis and event detection services operating in the deployment site platform. In this scenario, IoT-Stream can be used to semantically annotate information about the sensor devices and the data stream generated by them. It can also annotate applied analysis techniques, and events for emergency triggers, such as falls or persistent degradation of ambient air quality. When conducting pilots, the quality of information for data streams relating to consistency and reliability is an important requirement for providing technical feedback for the system, which is captured by QoI concepts [42]. As a distributed platform among different deployment sites, the discovery of data stream providers can be annotated using the iot-lite:Service, which in turn would enable cross-correlation Analytics among all users, groups, or services within the pan-European ecosystem. The number of streams, derived streams, stream observations, analytics techniques, and events from such an ecosystem is large, and therefore IoT-Stream can provide a lightweight and flexible approach to annotation.

To take a more concrete example for a use case such as daily activity monitoring that involves stream data analysis and event detection, the approach as defined in [43] can be taken, whereby the aim is to detect the level of activity in a home using a range of fixed sensor devices, such as PIR motion, door, energy consumption and pressure mat sensors. Each of these sensors generates a stream of raw observations which are individually fed to an analytics service that applies filtering and aggregation, although, in the case of occupancy, published stream observations are fed to another analytics service that applies rule-based reasoning to verify the stability of the reading over a period of one minute. The result of this is a derived set of streams which shows the summation of triggers over a period of one hour. The set of streams published by the first two analytics services are then fed into another analytics service that applies K-means clustering to the whole set of streams and produces two clusters which correspond to low activity and high activity. Events are then generated with a label that reflects either state.

**Figure 10:**
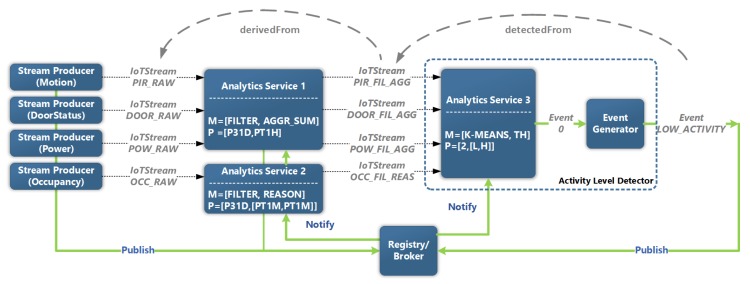
Annotation process for detecting indoor activity levels.

Figure 10 shows an excerpt of the instantiation of the system presented in Section 4, highlighting the main entities and their relationships. The figure also shows the annotations exchanged between system entities. The annotation process works in a similar manner as explained for Figure 8, with the exception that two analysed streams are derived from the raw streams, and both are fed into a third Analytics Service, which then generates an annotated Event with a label of either “HIGH_ACTIVITY” or “LOW_ACTIVITY”.

## 6. Tools and Applications

### 6.1. Data Analysis Tools

**Figure 11:**
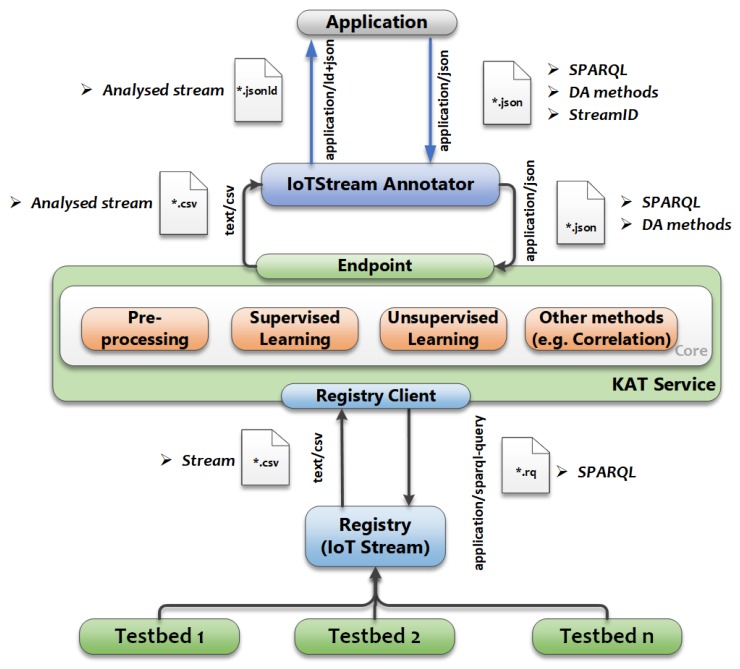
Analysed IoT-Streams using KAT service.

A system that consumes IoT data streams needs to employ some form of data analytics to handle the degree of volume, velocity, intermittency, irregularity, and dimensionality that comes with them. Libraries and frameworks for popular programming languages have enabled the creation of tools that handle data depending on its nature and the expected insights to be gained. Depending on the application, tools would involve some form of pre-processing, machine learning, or correlation. The output of such techniques can then be fed to enrich a semantic knowledge graph. The Knowledge Acquisition Toolkit (KAT) [38] web service provides such a facility that allows a consumer to first experiment with the remote IoT data sources with different cascades of methods to study which works best for them. By exposing a RESTful interface, KAT is able to query for data streams from a semantic IoT stream data store by accepting a SPARQL query with a predefined format for the output variables. In turn, the service will generate a new data stream based on the selected methods and their corresponding parameters. The new StreamObservations are then annotated and linked to a new IotStream, with the Analytics details employed, and then sent back to the Consumer. Figure 11 illustrates the process.

### 6.2. Crawling and Search Engines for IoT Data Streams

**Figure 12:**
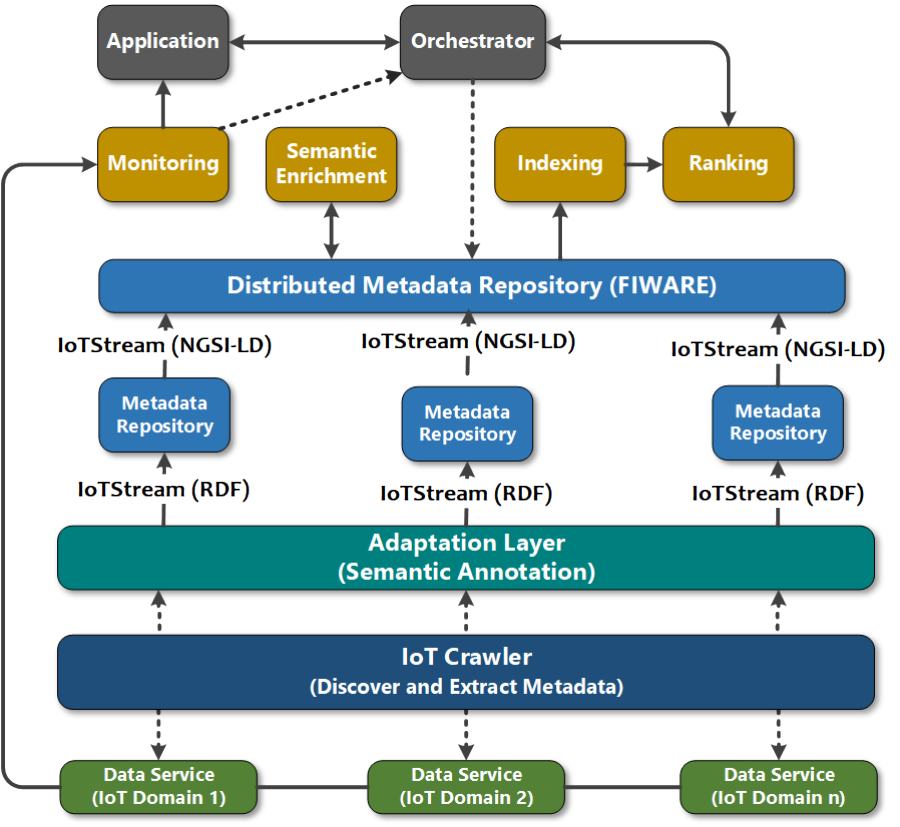
IoTCrawler Architecture with the use of IoT-Stream.

The IoTCrawler framework provides a crawler and search engine for discovering sources of multi-domain IoT data streams. The crawler extracts metadata from the data sources and pushes the metadata through an Adaptation Layer that semantically annotates the metadata as instances of IotStream, and is stored in an RDF metadata repository [44]. Metadata captured here include identification for the discovered IotStream and the sosa:Sensor generating it, and the the qu:QuantityKind being measured and the qu:Unit used for measuring. Geolocation information if provided will have the geo:Point containing absolute and/or iot-lite:relativeLocation information. The aim of the search engine is to provide Consumers with results that include information on how to reach and interact with the IoT data stream iot-lite:Service. The core system adopts a data broker from the FIWARE framework [45], which employs the NGSI-LD API [46]. This API uses a meta-model to encapsulate data models that are specific to a particular platform, in this case, IoT-Stream. The metadata collected is distributed among multiple brokers, known the Distributed Metadata Repository (MDR). The framework employs processing components that discover and analyze stream data from the Distributed MDR. One of the components, the Semantic Enrichment module, applies data analysis to evaluate the qoi:Quality, and is fed back to the MDR, and appended to the corresponding IotStream. The module also hosts pattern extractors that look for patterns in the data streams for a specified domain, which could include smart cities or smart homes, as described in Section 5. Certain patterns that are detected from the IotStream will then translate to Events, which are then pushed to the MDR. It is these Events that will serve as keywords for Consumers to use to search for data stream of interest, in addition to the data streams and sensors. To enable this, components relating to the search engine will index and rank IotStreams, sosa:Sensors, qu:QuantityKind and Events based on location and QoI metrics. This will allow Consumers to perform instant searches or subscribe to updates data stream services based on their preferences. Figure 12 show the architectural components in the IoTCrawler framework, and the annotation of streams using IoT-Stream.

## 7. Conclusions

Recently, semantics are becoming a key component in IoT applications to annotate sensor devices, but less likely the stream data they generate. Semantics provides the common language to interoperate between the heterogeneous sources of IoT data. However, semantics tend to model every detail of the domain, making the process of annotating and querying the stream data in heavy IoT environments, whereby data streams are numerous and continuous.

In this paper, we presented IoT-Stream, a novel semantic model for stream annotations and a system to effectively use the semantic model, which facilitates the implementation of IoT applications dealing with stream sensory data. We have developed the model according to the most recognized and state-of-the-art guidelines to develop semantic models, and especially guidelines for IoT environments, where the scalability and short processing time are essential. With these restrictions, we have created a lightweight semantic model fully compatible and as an extension of the well-known SSN ontology and its recent lightweight core SOSA. With the main concept, StreamObservation, with simple temporal and value properties, we have accelerated the queries to the stream model and have created the rest of the concepts needed for crawling and searching streams around this main concept. By doing so, we have improved the processing time of the stream queries. However, the ontologies need tools and guidelines to effectively use them and to facilitate the adoption of the data model. To overcome this lack of information, we propose a system architecture with the necessary entities to annotate and consume IoT-Stream annotated data. This system architecture will facilitate the adoption and replication of our proposal. Analytics processes are increasingly being exposed as web services, either as part of a domain-specific application or as a loosely-coupled microservice optimized for a dedicated analytical method. Therefore, our system architecture is based on those services, incorporating the annotation and consumption of IoT-Stream data.

We have proved the validity of IoT-Stream and the proposed system architecture through a series of real annotation scenarios. In each scenario, we have provided a detailed description of the entities/modules used and the relationship between them; i.e., we have instantiated the proposed system. The ontology is publicly available (http://iot.ee.surrey.ac.uk/iot-crawler/ontology/iot-stream) with dereferenceable concepts and available in several formats, some of them originally created from the field of ontologies, such as RDF and Turtle, and some of them adapted to ontologies from the field of Web development, such as JSON-LD (which are the fields of the potential developers using the ontology). This variety of formats will facilitate the adoption of the ontology from different developers with different backgrounds, and various applications, making interoperability easier. For fast adoption of the model, and, even for those not experts on ontologies, we have developed several accompanied tools, such as annotators, shown in the use cases, stream analyzers, and crawling and searching engines. We believe that our proposal would serve as an important reference for revising the analytics pipeline.

## Figures and Tables

**Table 1:** Namespaces and preferred prefixes from linked ontologies adopted for IoTStream.

Prefix	Namespace
iot-stream	http://purl.org/iot/ontology/iot-stream#
sosa	http://www.w3.org/ns/sosa/
iot-lite	http://purl.oclc.org/NET/UNIS/fiware/iot-lite#
qoi	https://w3id.org/iot/qoi#
qu	http://purl.oclc.org/NET/ssnx/qu/qu#
surrey-iot (individual)	http://iot.ee.surrey.ac.uk/living-lab/environmental/

## Abbreviations

The following abbreviations are used in this manuscript:

API	Application Programming Interface
CKAN	Comprehensive Knowledge Archive Network
C-SPARQL	Continuous SPARQL
CSV	Comma Separated Vector
EP-SPARQL	Event Processing SPARQL
GIS	Geographic Information System
GMM	Gaussian Mixture Models
IoT	Internet of Things
IRI	Internationalized Resource Identifier
JSON	JavaScript Object Notation
JSON-LD	JSON Language Description
KAT	Knowledge Acquisition Toolkit
LPR	Lagrangian Pattern Representation
M3	Machine-to-Machine Measurement ontology
MDR	Metadata Repository
NGSI	Next, Generation Service Interface
NGSI-LD	NGSI Language Description
NO2	Nitrogen Dioxide
OGC	Open Geospatial Consortium
PM	Particulate Matter
PIR	Passive InfraRed sensor
PROV-O	Provenance Ontology
QoI	Quality of Information
QU	Quantity and Units ontology
RDF	Resource Description Framework
REST	Representational State Transfer
SAO	Stream Annotation Ontology
SAX	Symbolic Aggregate Approximation algorithm
SOSA	Sensor, Observation, Sample, and Actuator model
SPARQL	SPARQL Protocol and RDF Query Language
SPARQLstream	SPARQL Stream
SSN	Semantic Sensor Network model
STARQL	Streaming and Temporal ontology Access with a Reasoning-based Query Language
SysML	Systems Modelling Language
TCP/IP	Transmission Control Protocol/Internet Protocol
W3C	World Wide Web Consortium
WADL	Web Application Description Language
